# Oxymatrine Inhibits Proliferation and Migration While Inducing Apoptosis in Human Glioblastoma Cells

**DOI:** 10.1155/2016/1784161

**Published:** 2016-11-13

**Authors:** Feili Liu, Baocheng Wang, Jiajia Wang, Xiaozheng Ling, Qifeng Li, Wei Meng, Jie Ma

**Affiliations:** Department of Pediatric Neurosurgery, Xinhua Hospital, School of Medicine, Shanghai Jiao Tong University, Shanghai 200092, China

## Abstract

Oxymatrine (OMT), an alkaloid derived from the traditional Chinese medicine herb Sophora flavescens Aiton, has been shown to exhibit anticancer properties on various types of cancer cells. In this study, we investigate the anticancer properties of OMT on human glioblastoma (GBM) cells and evaluate their underlying mechanisms. MTT assays were performed and demonstrated that OMT significantly inhibits the proliferation of GBM cells. Flow cytometry suggested that OMT at a concentration of 10^−5^ M may induce apoptosis in U251 and A172 cells. Western blot analyses demonstrated a significant increase in the expression of Bax and caspase-3 and a significant decrease in expression of Bcl-2 in both U251 and A172 cells. Additionally, OMT was found by transwell and high-content screening assays to decrease the migratory ability of the evaluated GBM cells. These findings suggest that the antitumor effects of OMT may be the result of inhibition of cell proliferation and migration and the induction of apoptosis by regulating the expression of apoptosis-associated proteins. OMT may represent a novel anticancer therapy for the treatment of GBM.

## 1. Introduction

Glioblastoma multiforme (GBM) is the most common, aggressive, and lethal primary brain tumor in adults and according to the U.S. Central Brain Tumor Registry accounts for 45.6% of all malignant brain tumors [1]. The current standard treatment remains gross total surgical resection followed by radiotherapy with concurrent adjuvant temozolomide (TMZ) [2, 3]. However, despite decades of GBM research, the median overall survival (OS) of GBM patients remains at just 8 to 14 months [4]. The difficulty in treating GBM has attributed its aggressive characteristics, including diffuse infiltration, rapid progression, resistance to radio- and chemotherapy, and ineffective drug delivery [5, 6]. Therefore, there is a great need for investigations into the mechanisms of GBM development as well as novel treatment strategies.

Oxymatrine (OMT; molecular formula, C_15_H_24_N_2_O_2_) is one of the main alkaloid extracts from the root of Sophora flavescens Aiton, an herb used in traditional Chinese medicine and known therein as Ku Shen. OMT has been reported to have various medicinal qualities, including antiviral [7], antifibrotic [8], anti-inflammatory [9], and antiarrhythmic [10] effects, and is also widely used in China for the treatment of chronic hepatitis B. Previous in vitro and in vivo studies have shown that OMT inhibits cell proliferation and induces apoptosis in various types of cancers [11–15]. OMT has also been shown to decrease the migratory ability of different cancer cell lines [13, 15]. In this study, we investigate the anticancer properties of OMT on human glioma cells and evaluate their underlying mechanisms.

## 2. Materials and Methods

### 2.1. Reagents and Antibodies

OMT (Shanghai Jinsui Biotechnology Co., Ltd., China) and TMZ (Sigam-Aldrich, USA) were dissolved in dimethyl sulfoxide (DMSO) and distilled H_2_O at a stock concentration of 0.1 M and then further diluted in culture medium to achieve OMT at 10^−7^, 10^−6^, and 10^−5^ M and TMZ at 100 *μ*M (positive control). Additional materials used included 3-(4,5-dimethylthiazol-2-yl)-2,5-diphenyltetrazolium bromide (MTT; Sigma Chemical Company, USA),an Annexin V-APC/propidium iodide (PI) apoptosis detection kit (BD Biosciences, USA), and a Cell Motility HCS Reagent Assay Kit (Thermo Scientific, USA). Mouse anti-human monoclonal antibodies specific for caspase-3, Bcl-2, and Bax were purchased from Cell Signaling Technology (USA), and the secondary antibody was obtained from Santa Cruz Biotechnology (USA).

### 2.2. Cell Lines and Culture

Human GBM cell lines U251 and A172 were obtained from the Shanghai Cell Institute Country Cell Bank (China). These cells were routinely cultured in Dulbecco's modified Eagle's medium (DMEM, Gibco, USA), supplemented with 10% fetal bovine serum (FBS, Gibco, USA) and 1% penicillin/streptomycin, at 37°C in a 5% CO_2_ incubator.

### 2.3. Cell Viability Assay

Cell viability was evaluated using an MTT assay. U251 and A172 cells were seeded into 96-well plates (500 cells/well) and cultured at 37°C for 24 hours. The cells were then exposed to OMT, TMZ, or 1% DMSO at the aforementioned concentration levels for 24 hours. Subsequently, 20 *μ*L of MTT (5 mg/mL) was added to each well and they were incubated for 4 hours. The medium was removed, the MTT-formazan precipitate was dissolved in 150 *μ*L of DMSO, and absorbance values were measured at 570 nm.

### 2.4. Transwell Migration Assay

Transwell migration assays were performed to determine the effects of OMT on GBM cell migration. U251 and A172 cells (500 cells/well) were seeded into the upper chamber of a polycarbonate transwell plate (8 *μ*m pores size; Corning, USA) in 300 *μ*L of a serum-free medium with OMT, TMZ, or 1% DSMO at the above concentrations. 800 *μ*L of DMEM with 10% FBS was then added to the lower chamber as a chemoattractant. The plate was incubated for 10 hours and nonmigrated cells were removed from the upper surface of the filter with a cotton swab. The cells that migrated to the lower chamber were stained with 0.1% crystal violet for 15 min at room temperature and were then photographed and counted under high magnification.

### 2.5. High-Content Screening Assay

Cell motility was analysed using a high-content screening assay. GBM cells in log phase were seeded into 96-well plates (500 cells/well) and coated with Collagen I and blue fluorescence beads. The cells were cultured in a medium containing the aforementioned concentrations of OMT, TMZ, or 1% DMSO for 10 hours. Cell motility was then measured using the ArrayScan HCS Reader (Thermo Fisher Scientific In, USA) by following the manufacturer's protocol.

### 2.6. Annexin V-FITC/PI Double Staining

Annexin V-FITC/PI double staining was utilized to quantify apoptosis. Cells were seeded in six-well plates (2 × 10^5^ cells/well) and exposed to the same concentrations of OMT, TMZ, or 1% DMSO at the above-mentioned concentrations for 24 hours. The cells were then stained and analysed using an Annexin V-FITC/PI double-fluorescence apoptosis detection kit.

### 2.7. Western Blot Analysis

Proteins were extracted from U251 and A172 cells following treatment with OMT, TMZ, or 1% DMSO at the above-mentioned concentrations for 24 hours. Proteins were then separated using sodium dodecyl sulfate polyacrylamide electrophoresis gel (SDS-PAGE) and transblotted onto polyvinylidene difluoride membranes at 100 V for 1 hour. Membranes were blocked with 5% skim milk powder in tris-buffered saline containing 0.1% Tween-20 for 1 hour at room temperature and then incubated with caspase-3, Bcl-2, and Bax antibodies (1 : 1,000) overnight at 4°C. *β*-actin was used as an internal control. Membranes were then incubated with goat anti-mouse secondary antibodies for 60 minutes at room temperature. Protein bands were then observed in an imaging system.

### 2.8. Statistical Analysis

Statistical analysis was performed using GraphPad Prism version 5 (GraphPad Software, Inc., USA). All data is expressed as mean ± standard deviation. Multiple comparisons between groups were made by analysis of variance (ANOVA) and Tukey's tests for post hoc testing. Statistical significance was considered when *P* < 0.05.

## 3. Results

### 3.1. OMT Decreases the Viability of GBM Cells

**Figure 1:**
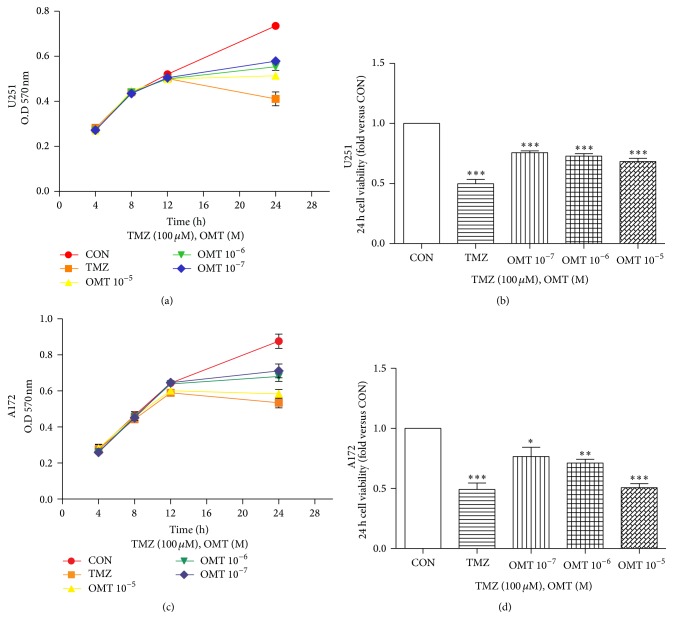
Oxymatrine decreases viability of GBM cells. (a, b) U251 cell and (c, d) A172 cell viability measured by MTT assay. Cells viability was normalized to that of the negative control (CON) group. ^*∗*^ *P* < 0.05, ^*∗∗*^ *P* < 0.01, and ^*∗∗∗*^ *P* < 0.001.

MTT assays demonstrated the ability of OMT to efficiently inhibit the growth potential and viability of GBM cells. Each concentration of OMT applied significantly decreased the viability of both U251 and A172 cell lines (Figure 1). The positive control, TMZ, also demonstrated significant inhibition of GBM cell viability. These data suggest that OMT may efficiently inhibit viability of GBM cells.

### 3.2. Effect of OMT on GBM Cell Apoptosis

**Figure 2:**
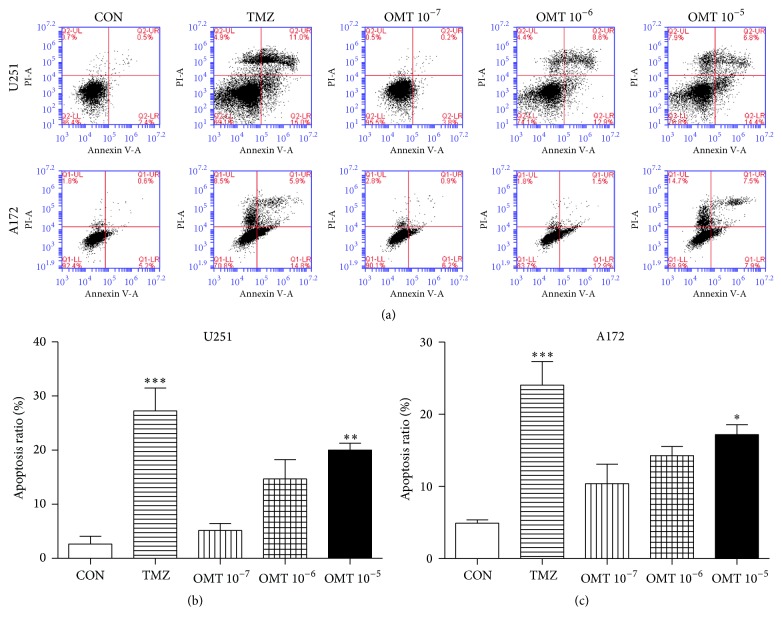
Oxymatrine induces apoptosis of GBM cells. (a) Representative flow cytometry analysis of U251 and A172 cell apoptosis costained with Annexin V/PI and treated with different concentrations of OMT for 24 hours. (b, c) Rate of apoptosis in U251 and A172 cells by treatment group compared with the negative control (CON) group. ^*∗*^ *P* < 0.05, ^*∗∗*^ *P* < 0.01, and ^*∗∗∗*^ *P* < 0.001.

**Figure 3:**
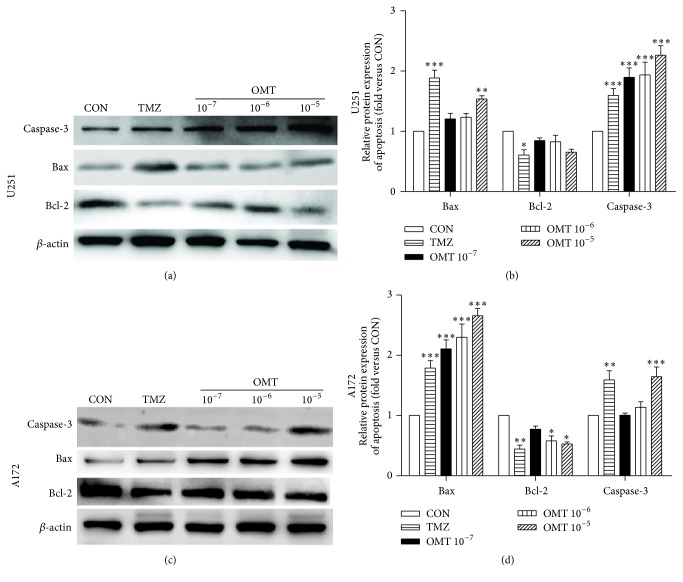
Oxymatrine induces apoptosis of GBM cells. (a, c) U251 and A172 cells treated for 24 hours with DMSO and indicated concentrations of OMT or TMZ. Expression of caspase-3, Bax, and Bcl-2 was analysed by Western blot. (b, d) Expression analyses were normalized to that of the negative control group (fold versus CON). ^*∗*^ *P* < 0.05, ^*∗∗*^ *P* < 0.01, and ^*∗∗∗*^ *P* < 0.001.

Flow cytometry analysis showed that treatment with OMT at a concentration of 10^−5^ M resulted in a statistically significant increase in GBM cell apoptosis (Figure 2). Western blot analysis revealed that expression of Bax and caspase-3 increased, whereas expression of Bcl-2 decreased (Figure 3), indicating that treatment with OMT may promote GBM cells apoptosis by regulating the expression of apoptosis-associated proteins.

### 3.3. OMT Inhibits Migration of GBM Cells

**Figure 4:**
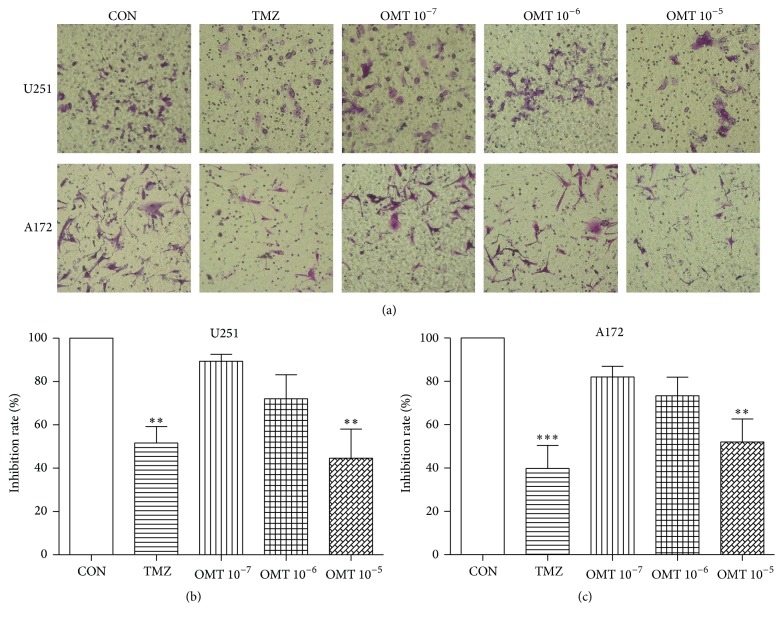
Oxymatrine inhibits migration of GBM cells. (a) Effect of OMT on U251 and A172 cell migration was assessed using a transwell migration assay. (b, c) Quantitative analysis of the inhibition rate (%) of U251 and A172 cell migration normalized to that of the negative control (CON) group. ^*∗∗*^ *P* < 0.01 and ^*∗∗∗*^ *P* < 0.001.

**Figure 5:**
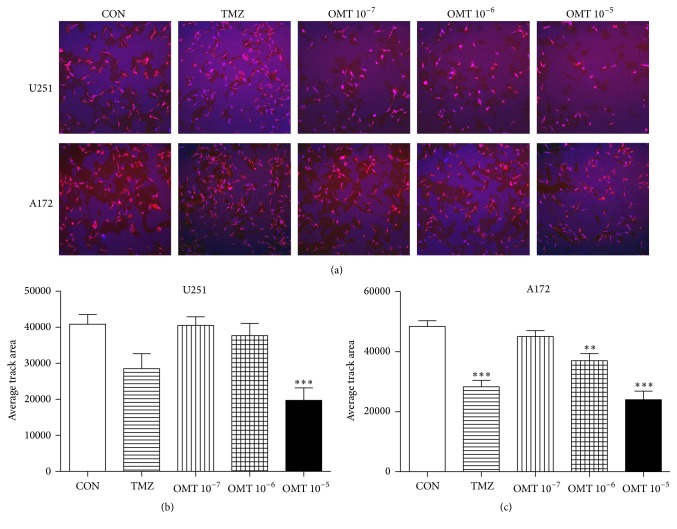
Oxymatrine decreases GBM cell migration. (a) Effect of OMT on U251 and A172 cell migration by high-content screening assay. (b, c) Quantitative analysis of the average track area of U251 and A172 cells normalized to that of the negative control (CON) group. ^*∗∗*^ *P* < 0.01 and ^*∗∗∗*^ *P* < 0.001.

Transwell assays revealed that OMT at a concentration of 10^−5^ M could significantly inhibit the migration of both U251 and A172 cells (Figure 4). Additionally, high-content screening assays showed that OMT at a concentration of 10^−5^ M significantly reduced the average track area of U251 cells compared to the control group. The track area of A172 cells was also significantly reduced in a dose-dependent manner (Figure 5). Collectively, this indicates that OMT may inhibit the migration of GBM cells.

## 4. Discussion

OMT has been extensively investigated for antitumor effects against various cancers [15], including lung [12], gastric [13], pancreatic [11], and breast [14]. However, to the best of our knowledge, the antitumor effects of OMT on GBM have yet to be fully investigated. In the present study, we treated U251 and A172 human GBM cells with various concentrations of OMT and found that OMT inhibited cell proliferation and migration and induced apoptosis.

Abnormal cell proliferation is a key element of tumorigenesis and thus inhibiting cell proliferation is considered an effective avenue for the development of novel antitumor therapeutics. We found, via MTT assay, that OMT significantly inhibits cell proliferation in both U251 and A172 cells. These results support previously published findings indicating that the main components of Sophora flavescens Aiton have an inhibitory effect on C6 cell proliferation [16, 17].

Apoptosis, characterized by DNA fragmentation, is a known defense mechanism against tumor formation [18] and is mediated by two signaling pathways, the intrinsic (mitochondrial) and the extrinsic (death receptor) [19, 20]. The Bcl-2 protein family, including antiapoptotic and proapoptotic proteins, such as Bcl-2 and Bax, plays an important role in the intrinsic apoptotic pathway and mediates the activation of downstream caspases [19, 21]. It is widely understood that caspase-3 is an essential terminal caspase that executes apoptosis and eventually leads to DNA fragmentation in the mitochondrial pathway [19, 21]. Therefore, induction of apoptosis may represent a potential approach for antitumor therapy [22], and there is an increasing body of evidence that OMT may induce apoptosis in a number of cancer cell lines [11, 12, 15, 18, 23–26]. The data presented herein adds to this body of evidence by demonstrating that OMT induces apoptosis, decreases expression of Bcl-2, and concurrently increases expression of Bax and caspase-3. Collectively, these findings strongly suggest that OMT exhibits antitumor properties, possibly by mediating apoptosis.

The poor prognosis associated with gliomas is partially attributed to the infiltration of GBM cells into the surrounding normal brain parenchyma. This diffuse infiltration often complicates or prevents complete surgical resection and increases the risk of tumor recurrence [5, 27]. Thus, multimodal treatment strategies, including antiproliferative agents, are necessary for GBM [28]. The antimetastatic potential of OMT on GBM cells, as demonstrated herein, should be considered for further study as an anticancer agent.

## 5. Conclusions

OMT demonstrates significant antitumor properties on GBM cell lines. These effects may be a result of the inhibition of cell proliferation and migration and the induction of apoptosis by regulating the expression of apoptosis-associated proteins. Further in-depth investigations are necessary to fully determine these underlying mechanisms and in vivo studies are needed to confirm the anticancer activities of OMT. We believe that OMT may represent a novel anticancer therapy for the treatment of GBM.
